# American Academy of Dental Science

**Published:** 1891-08

**Authors:** 

**Affiliations:** American Academy of Dental Science


### AMERICAN ACADEMY OF DENTAL SCIENCE.

THE regular monthly meeting of the American Academy of Dental Science was held in the Boston Medical Library Association rooms on April 1, 1891, Vice-President Brackett in the chair.

The paper for the evening was read by William Barker, D.D.S. Subject, "A Discussion of the Fee Problem."

(For Dr. Barker's paper, see p. 533).

### DISCUSSION.

*Dr. Brackett.*—Gentlemen, you have heard the paper of Professor Barker, showing plainly, what some of us have appreciated before, that he is a student of social problems. He is especially known in Rhode Island as a profound student of social and economic questions, and he has made this the basis of a special application which certainly has been most interesting and suggestive for us. The paper is open for discussion.

*Dr. Fillebrown.*—I was much interested in Dr. Barker's paper. I did not quite understand, however, how one point would be met. It could be stated thus: Persons who may be well known to us, and whom we may desire to help, come into our office having some trouble with the teeth; we cannot allow them to suffer pain,—something must be done for them, but they have no means to pay, and we know it. The operation is going to take as much time as if we were paid for it, but we cannot turn them out and let them suffer. For humanity's sake we are bound to do something to alleviate that pain, and we cannot do it unless somebody else pays for it. This is an extreme illustration, but I did not hear such a case touched upon in the paper, and would like to have it explained.

There is another point, but perhaps that is covered by the idea of cost. One man is naturally much more skilful than another,—he can do an operation very much quicker, easier, and the results are better than those of another man who has to give twice the time for the same work. In the case of the skilful man, on account of his natural quickness and intelligence, the total cost of time and materials might be represented by five dollars, while in the other case the cost of it might be represented by twenty dollars. Now, ought the one who has performed the same amount of service with less discomfort for the patient accept a sum which represents the cost in time to him?

I am heartily in sympathy with the working of the social problem and the bringing it into practical use, but one cannot go to extremes while the general average of the world is so far behind in the practice of the system. I would like to hear how socialists, through social glasses, view the question of the charitable work that we are called upon to do. It seems to me that all our hospitals, dispensaries, and so forth, are based upon the supposition that the rich are quite willing to pay the fees that shall enable each man to do his share towards helping those who are less favored.

*Dr. Williams.*—The matter of charging by time is of modern origin, and many dentists make it an absolute gauge for fees. It was established about the time of the war, when things began to boom generally. I have never brought myself to making that the main consideration, for several reasons. In the first place, I have always believed in the fact that Dr. Holmes once stated, that the most healthy man does not feel equally well at all times in his life. Therefore, if you try to give the same amount of effort every day, you do violence to your constitutional stock,—you have to draw on your principal, as it were. Bearing this fact in mind, I have made it a rule to work as I feel. I would not feel as the plumber or the locksmith, that I was obliged to do just so much work because I was paid for so much time, but I would do the thing well if it took me twice the time, and I would charge for what I did. In some cases I have charged about as much as the highest fee Dr. Barker mentions for about half an hour's work, and in other cases my charge was probably not more than the lowest fee. I calculate mainly on what is done for the patient, taking the matter of time as a secondary consideration. I should not, as an orist, charge like some other specialists. For instance, I have known of a case where an aurist charged ten dollars for painting the back of the ear with iodine. It could not have taken much time,—I should think not as much as to take out a tooth, which I would charge a dollar for. It has never been my custom to make just so much every hour of my time, and I get along better mentally, physically, and, I think, more conscientiously than if I charged only by the time.

*Dr. Fillebrown.*—I made it a point not to antagonize the speaker in any way, but to draw forth a still further explanation of a part of the problem that bothers me. I should be glad to see the condition of society described in "Looking Backward" entirely realized here,—we could all of us live easier and better as far as our physical comforts are concerned, though we cannot tell what the effect would be on the moral and intellectual health. I heard of a case to-day that illustrates one phase of what I was alluding to. A gentleman told me of a friend of his who had had considerable dentistry done. At one time he was under the care of a dentist for more than a year for the treatment of a single tooth, and had been in trouble all the time. The patient then called on another dentist, and within two or three visits the tooth was cured,—made entirely healthy, and has remained in a healthy condition ever since. Now, if you are reckoning by the cost, the first fees were too large for the benefits which accrued; if estimated by the amount of good done, in the second case the fee was very small. There is the trouble in all these things. It seems to me that, under the present constitution of society, the principles laid down in the paper cannot be carried out. If we would all join hands at once, something might be done, still I don't see how we could overcome the question of natural aptitude. Under certain conditions one man might be entitled to ten dollars and another would only be entitled to ten cents. It was stated in the paper that corn, beef, and pork cost alike to each one. That is not true; we know perfectly well that the millionnaire buys more delicate goods and pays more for his beef, canned goods, and all articles of food than he who is earning but two dollars a day. He pays more for his cooks, and has his food better served than persons of moderate means. He may even pay ten thousand dollars a year for a cook, where most pay but a few dollars a week. There are many things which go to increase the cost of his food, so that there is a difference in the cost of even the simple things that go on his table.

*Dr. Allen.*—There is a principle at stake here which every one should recognize. I feel that each one should know for himself what his time and his services are worth, and that we should seek to enlighten our patient in this regard. As a rule, we are apt to gauge the value of our services by what we can get for the same. For instance, a young practitioner may not feel that his services would bring as much as one who has been in practice for twenty years, or longer. It seemed to me that the simplest way of getting at the point is for each one to determine for himself what he is willing to work for,—the sum that a day's labor ought to yield,—and having determined that point, let us gauge our fees accordingly. I am perfectly willing to work upon that basis. I believe that we can work with much more justice to ourselves and to our patients if we make a definite charge for the time the service occupies, the cost of the material used should not be specially considered (except in artificial dentures) as it is very trifling compared with the fees charged.

Regarding charitable work, if I give an hour's service I give it in the name of charity. I do not ask or expect any one else to pay for the bestowal of my charity. I will work the remainder of the day, and apply the net proceeds to myself.

*Dr. Clapp.*—One remark of Dr. Allen's has great force in this connection, and that is, that each man must, so to speak, be a law unto himself. I do not believe that there is a business, a specialty, or a profession, or any occupation in the world that requires so much uprightness and force of character to stand on the top plane of possibility as the dental profession, and there is no man living, outside of the operator himself, who can properly say what his fee should be. There is no other man that knows the circumstances, and when we come to gauge and to determine the fees for dental operations by any fixed law, or by any analogy of reasoning, I do not believe it is possible to equitably do it. Some time ago I went to a physician to pay a bill, and at the same time that I was in his office I was troubled with a slight affection of the throat. He is one of our foremost practitioners here in the city, and I asked him to look at my throat. I stepped into the light and opened my mouth. He looked into my throat and said, "Humph!" then turned and wrote a prescription, and handed it to me. The whole thing did not exceed one minute and a half, and his fee was three dollars. Now, I did not pay him for the time he occupied in looking into my throat and writing the paper,—I paid him for his twenty-five years' experience. And, to my mind, he gave me greater service in that minute and a half than many another man would after studying my case an hour. The matter depends largely upon one's own personal experience and one's own conscientious application to the question of fees as well as to means and methods of work.

*Dr. Grant.*—I do not know that I can add anything to what has already been said. I am very much interested in Dr. Barker's paper, and the thought occurred to me, which has already been so well stated by Dr. Allen and Dr. Clapp, that there cannot possibly be any other standard for fixing fees except the man's own judgment in the matter. He has of course many things to consider,—first, what his education has cost him; then the class of patients he can command, and also his years of active life. I do not know exactly how a man can fix the standard of his professional services, it depends a great deal upon his motive in entering the profession, whether to gain fortune, or simply for what is the ideal plan,—of doing the best he can for humanity and getting simple remuneration that shall give him comfortable life. Every laborer is worthy of his hire, but while many men put much time and comparatively small capital in cash into their profession, of course many others put both, and then there is the interest on time that he spends in perfecting himself and the interest on actual capital invested to be taken into consideration. Of course, the man who gives both time and money to his profession cannot afford to work on the same plane as the man who only gives his time. I think the principal element which is taken into consideration in adjusting fees is the class of patients that are attracted to a man either by his professional reputation or by his social standing. It seems impossible that there should be a general plane upon which all dental fees could be gauged, each one of us must do as our conscience and our foresight in the matter of temporal affairs shall dictate.

*Dr. Williams.*—Perhaps some of you may have heard of the curious way in which a dentist in one of the suburbs of Boston used to charge for his services. He charged according to the number of sheets of gold he put in a tooth, and, of course, the effect of it was for him to see how much gold he could pile on, and he used to make some very peculiar structures.

*Dr. Brackett.*—I do not think it is essential, because we perform a gratuitous operation for a patient who needs it, that we should charge for that operation some one who is more than able to pay. I think the remarks of Dr. Allen—"If it goes as charity it should really be charity"—are most commendable. In this particular, although we do a great deal for which we receive no compensation, it is little compared with what those of other professions, especially physicians, do; and it has been urged against our claims to professional standing that we have not done the amount of gratuitous service that men of broad cultivation and higher plane might well do for the advantage of humanity and the world.

*Dr. Smith.*—It may be unfortunate, from a purely scientific point of view, that fees have anything to do with our work. If we were all born of wealth, and could practise our calling merely for the love of it, we would not have this question to contend with; but with things as they are, it is a matter largely of supply and demand, and the fee must be limited to that demand. For instance, if a practitioner finds his time taken from early morning until night, with others begging him to work on the Sabbath, he can with justice say to himself, "My fees shall be a little more than they have been. There is a demand for my services by people who have the money and are willing to pay for them." I have had people say to me, "Dr. So-and-so charges fifteen dollars an hour for his services, don't you think he is a robber?" I would say, "Not at all; I suppose he is now obliged to refuse appointments with people who would pay him twenty dollars an hour if he could accommodate them." I think our fees must be limited almost entirely by the demand upon our professional services.

It has been said that the dental schools, the hospitals, and the infirmaries take away, in a measure, the opportunity for young practitioners to gain an income from their practice, which is very true; but it must not be understood that we are opposed to the hospitals and infirmaries. They should be conducted, however, in such a manner as to cater only to the strictly charitable patients. The demonstrator, I think, should have the power to make a distinction between those who can afford to pay and those who cannot, and when a woman goes to a dental school wearing diamond ear-rings, seal-skin sack, and eight-button gloves, it is time she went to some office and paid a fee.

With regard to charity work, it is much harder for us to do this work and not suffer more for it than any other profession. A medical practitioner can see a number of patients in a short time, many of the cases being evident to him at a glance, and requiring but a minute and a half of his time, as the one cited here to-night. What can a dentist do in a minute and a half that is charity? If he fills a tooth, it takes time; if he treats a tooth, it takes time; and if he tries to do much of this work, he finds he has used up a large share of his time, and his time is his stock in trade. A lawyer can give his advice and do charity work and his income does not feel it, and there are other professions which do not suffer financially from the charity work which they see fit to give. Physicians suffer largely from our hospitals and charitable institutions by the richer people taking advantage of them. I was told by a noted surgeon that wealthy patients will go to the hospital and take a private room and have the services of the best surgeons, and it costs them nothing for the surgeon's services, they merely pay the rent of their room, the surgeon being obliged to give his services because of the position he holds. I understand that it is becoming a common practice for rich people to go to hospitals where they have the services of the physician and nurse free; perhaps they think they are better treated. The physicians have a sliding scale of prices; for instance, the fee charged by a noted oculist for the removal of a cataract for a very rich patient was five hundred dollars; for a patient in moderate circumstances his charge would be two hundred and fifty dollars; for a person in less than moderate circumstances, one hundred dollars, and even less than that, so in that case the rich pay for the poor. To a certain extent I think that is just. The oculist may put himself on this ground: that his fee for the removal of a cataract is five hundred dollars; every dollar which he discounts from that is to that extent charity work.

There is another point of which I wish to speak, and that is, that a fee should be charged for everything we do, unless we make it a charity case and charge nothing. It is quite common among dentists to make an examination of the teeth, which, if properly done, takes fully half an hour, and yet it is a common practice to make no charge for it, in the expectation that the patient will have to pay for it later on. I say that is unprofessional. I say that every operation—and an examination, I hold, is one of the most important of operations—should be charged for.

In regard to whether we should base our charge upon the minimum or maximum basis of time,—I don't think either is correct; it depends entirely upon the patient. For instance, if I am performing an operation for a very nervous patient, and it takes me two hours to do what I might do for another in an hour's time, I hold that patient should pay me just twice as much. I sympathize with the lady on account of her peculiarly nervous temperament and know that she is not to blame for it, yet I cannot afford to suffer financially for her misfortune.

*Dr. Barker.*—Of course, Mr. President, for me to say anything is only to say in a different way that which I have already said in the paper. But before I attempt to go over the ground which I attempted to cover in the paper, I want to say one word with reference to myself. There seems to be an idea prevalent that I

am a socialist. I am not a socialist, nor am I a nationalist, probably no more so than most or any of you here. I simply believe in justice. I believe that one of the first principles which should govern any person after they have arrived at adult age is the principle that they are bound to take care of themselves. God gave us all a stomach which needs supplies of food, and with the stomach he gave us two hands to supply the needs of the stomach, and absolutely no one but ourselves ought to be expected to or be called upon to supply those needs, provided we have health and strength. And let me say incidentally that if conditions were as they should be, there would be an opportunity, at least, for every one to employ the hands for the satisfaction of the needs of the stomach, and we should not find a large class in this community—as we find in every community—not only supplying their own needs, but supplying the needs of a large class of drones, which we may liken to barnacles on a ship, aiding in no way its passage over the waves, but rather retarding its progress. Of course, this question resolves itself at bottom into a great moral question. Now, if you admit that when a man arrives at adult age he is bound to take care of himself, that he may not rightfully call upon anybody else to take care of him,—if then he is bound to supply his own needs, and may not rightfully be called upon to supply any but his own needs, will some one please tell me how we can fix on anything but cost as the limit of price which he is to give to his neighbor in exchange for something which the neighbor gives him. I admit that it is difficult to determine the absolute ideal in any given case, but let us suppose that it has cost me in time, effort, material, and repugnance to overcome something which Mr. Smith wants more than he wants the coat on his back. He has an extra coat, which he thinks will do him just as well. I like that coat, and a barter is arranged between Mr. Smith and myself. Suppose we make an exchange. If I am to get in that exchange a coat which cost him more than twenty, why I have been getting something for nothing, and manifestly, I have been making Smith contribute to my support, whereas I ought to support myself. We become perplexed in thinking over this question, simply because, under present conditions,—and these same conditions have existed for many centuries and are likely to exist for some time to come,—this principle has not been recognized. We all of us say if we get a hungry man who will pay us a good price, we will take it from him, and we practise it too. In spite of the illustration offered to-night to prove that the rich man pays more for his needs than the poor man, it is not a fact. The grocery where I trade is patronized by men of all classes, and the wealthy men do not pay a penny more for the sugar, the flour, or the beef they use than any of the others. And they ought not to. What right has the grocer, the dentist, or the doctor to say what the ability of the purchaser is? What right have I to say to the seeker of my services that he shall pay a fee proportionate to his ability to pay? It is none of my business, gentlemen, what the ability of the purchaser is, it is none of your business. You may estimate the cost of your services, and the would-be purchaser, if he is a wise man, will place his own estimate upon their value to him, and if the cost seems to him to be greater than the value, he will not purchase. And now, because we have a class in every community who have amassed colossal fortunes, and are rich beyond the dreams of avarice, because they have ignored this cost principle, and they fall into your hands, you say you will recover your loss by getting all you can from them. Because he has been a great bandit, you will be a lesser bandit, and think it is all right to take from him a part of that which he has taken from others. The ideal thing is for every man to take care of himself, to live upon the rewards of his own industry, keeping his hands entirely off of that which his own industry did not produce. But, some one will say, am I to starve? And, as I said in my paper, a man who should undertake to do business upon ideally equitable terms where no obligation to reciprocity was recognized manifestly would have to go to the wall, and when you come to look at this question closely it resolves itself down to this: How far may I violate a correct principle in order to maintain my life? As I told you in my paper, conservation of energies is essential. Believing that I have no right to take from a patient in fee one penny more than the service has cost me, I may rightfully, however, include in the reckoning the cost of material and time expended directly in it, and a proportionate part of the time, thought, and study expended in my preparatory student days, and something for repugnance overcome, until finally, as I said before, it comes down to a question of casuistry,—How far am I willing to conform to a principle which seems correct? One of the great troubles comes in the belief or conception which most people have, that they ought, during the years of their active life to accumulate wealth enough to erect a barrier between themselves and that contingency, which none of us like to contemplate, namely, the poorhouse, and so they say, if I can find a rich man here and another one there, I will plunder them by the ordinary means, and I will use my plunder to erect this wall or barrier. My object in this paper is simply to point out a principle, leaving each man himself to be the judge as to how he shall apply it in his own practice. We sometimes hear that a certain dentist gets twenty dollars an hour for his services. Did you ever really think what it meant? The majority of people, right here in the city of Boston, working for a living, cannot command on an average more than two dollars a day for their labor, and yet some dentist, because he has spent some time and money in educating himself, thinks he has a right to appropriate in one hour what that man would earn in ten days. I simply say that such a fee as that violates the reasonable correspondence between cost and price. I do not undertake to say just where the line lies, no man can say that. I have an item in my note-book, clipped from a paper, which says that a dentist in New York took five hundred dollars in one fee for a service which occupied but ten hours of his time. What items of cost were there included to make his services worth fifty dollars an hour? I thought at the time I first saw it that it was infernal robbery, and I think so still, and I believe that any man who is willing to support himself and wants to come honestly by everything that he gets cannot charge any such fees.

Some half a dozen of you have summed it up, now let me sum it up. You seem to think when you are disposing of your services to men of varying means, some of them millionnaires and some of them poor men, that you have a right to take what they can afford to or can be made to give you. Let us place ourselves in the position of the buyer who needs our services, and who says, rather than go without them, he will pay five hundred dollars, and we can perhaps better see the injustice of making one's ability to pay, or the value of the service, a measure of its price. Our steam- and horse-railroads, our theatres and our merchants, from an A. T. Stewart to the peanut-vender under the shadow of his war-horse, have the same price for a like service to rich and poor alike, and there is no principle which can govern any other course. To get what you can is not principle. It is something suggestive of cannibalism.

In disposing of our services let us ask, What has it cost me? and keep out of sight, so far as possible, the question, How much can he afford to pay, or be made to pay? Self-preservation and the continuance of civilized institutions will sooner or later force society to adopt some such principle as we have presented. In the mean time let each man hew as near the line as, under all the circumstances of his environment, he can, and all the sooner shall the drones be driven from the hive, all the sooner shall labor and industry have its full reward, and idleness and inefficiency likewise receive their rewards, and so civilization will be perfected.

Subject passed.

*Dr. Fillebrown.*—I have here a record of twenty-two consecutive cases in which I have used the dental thermo-obtunder. If there are any members here who have not seen this instrument, I will say that it is a little cylinder which holds a small cartridge. The cartridge contains a wick which is saturated with alcohol. Beyond the cartridge is a copper bulb which is heated, and beyond that is a tube which carries a small spray of alcohol vapor into the tooth. The temperature conveyed by the alcohol vapor is about 110° F., and it usually takes about sixty seconds to obtund the tooth.

Eighteen of my cases the patients pronounced completely successful. Four cases were only partially successful, but the operations were made more endurable by the use of the obtunder. Of the four cases, two were much benefited, but suffered considerably in the application of the remedy. One case suffered much in the application, though it was made gradually, and seemed to get no benefit at all.

*Dr. Andrews.*—I have had one of these appliances for a week, and have had no occasion to use it.

*Dr. Ainsworth.*—It is remarkable, when a person has the instrument, how seldom he will require to use it.

*Dr. Allen.*—I suppose many of you know I have used that instrument almost from its first inception. Mr. Small brought a very crude form of it to me shortly after he brought it to the notice of the profession, and I used it with excellent results. He subsequently furnished me with a smaller instrument, which does the work equally well and can be more easily handled. I do not have occasion to use it every day, but I have been very successful with it, and I have yet to see the first case where permanent injury has been done by it.

*Dr. Fillebrown.*—Sometimes, it seems, the presence of an instrument prevents the need of it, but we also find that there are many times when we can use it effectively. I did not say that these cases came right along consecutively, or that I need it in every operation, but I have taken the cases as they occurred, omitting none, and brought them all together. So far as I have gotten in the matter, it seems to be a very desirable thing to have. The success of this, as other instruments, depends upon the faith and skill with which an operator uses it, and the determination he may have to thoroughly understand it.

*Dr. Ainsworth.*—Perhaps I have not put myself clearly before the society in this matter. I believe in the instrument and believe it will accomplish a great deal. I have for a long time, as many of the rest of you probably have, used alcohol in various ways, both hot and cold, but this instrument seems very much superior to anything that I have seen or thought of. I consider it a valuable acquisition.

*Dr. Barker.*—Our President, Dr. Seabury, requested me to bring some aluminum foil to the Academy this evening, and such of you as wish can take some of it and experiment with it. It has been recommended as a filling-material, but he says that he thinks this particular make is too fine for practical purposes, but perhaps a little heavier make than this might be used with advantage.

WILLIAM H. POTTER, D.M.D.,
*Editor American Academy of Dental Science.*

